# Protein interaction sentence detection using multiple semantic kernels

**DOI:** 10.1186/2041-1480-2-1

**Published:** 2011-05-14

**Authors:** Tamara Polajnar, Theodoros Damoulas, Mark Girolami

**Affiliations:** 1 School of Computing Science, University of Glasgow, Glasgow, UK; 2 Department of Computer Science, Cornell University, 14850, Ithaca, NY, US; 3 Department of Statistical Science, University College London, London, UK

## Abstract

**Background:**

Detection of sentences that describe protein-protein interactions (PPIs) in biomedical publications is a challenging and unresolved pattern recognition problem. Many state-of-the-art approaches for this task employ kernel classification methods, in particular support vector machines (SVMs). In this work we propose a novel data integration approach that utilises semantic kernels and a kernel classification method that is a probabilistic analogue to SVMs. Semantic kernels are created from statistical information gathered from large amounts of unlabelled text using lexical semantic models. Several semantic kernels are then fused into an overall composite classification space. In this initial study, we use simple features in order to examine whether the use of combinations of kernels constructed using word-based semantic models can improve PPI sentence detection.

**Results:**

We show that combinations of semantic kernels lead to statistically significant improvements in recognition rates and receiver operating characteristic (ROC) scores over the plain Gaussian kernel, when applied to a well-known labelled collection of abstracts. The proposed kernel composition method also allows us to automatically infer the most discriminative kernels.

**Conclusions:**

The results from this paper indicate that using semantic information from unlabelled text, and combinations of such information, can be valuable for classification of short texts such as PPI sentences. This study, however, is only a first step in evaluation of semantic kernels and probabilistic multiple kernel learning in the context of PPI detection. The method described herein is modular, and can be applied with a variety of feature types, kernels, and semantic models, in order to facilitate full extraction of interacting proteins.

## Background

Proteins are the principal engine enabling chemical reactions in a cell, and, as such, are of great interest to biologists studying life on the molecular level. Part of the proteins' functionality depends on their interactions with each other. Information about these interactions is paramount to the understanding of pathologies, diseases, and treatments. The principal observations of interactions are made through biological experiments [1], whose results are reported in peer-reviewed biomedical journal articles. Protein-protein interactions (PPIs) are then found by researchers through various search engines indexing these specific articles. In text, a PPI is a relation between two protein entities linked by an action descriptor, which is usually either a verb, or a present (-*ing*) or past (-*ed*) participial adjective (e.g. *activate*, *activating*, *activated*). A relationship is difficult to describe using a query; therefore, current state-of-the-art search engines are not well suited for this task. In addition, *ad hoc *query-based searches are more appropriate for temporary information needs, not persistent ones [2]. For research tasks such as pathway construction or population of PPI databases such as KEGG [3], MIPS [4], or BIND [5], PPI extraction becomes a continuous process. Consequently, PPI detection and extraction have become one of the primary goals of biomedical text mining (TM) [6]. The aim is to develop applications that will enable habitual PPI searchers to find interactions without having to specify pairs of proteins or manually scan large amounts of text.

Automatic protein interaction detection can be useful in several different scenarios. There are, therefore, many different approaches for information extraction in the biomedical text. Some applications are geared towards helping with automatic population of interaction databases [7,8], while others aim to support a wide variety of users by bridging the gap between the search engines and highly customised relation extraction software [9-12]. Different approaches to PPI detection can be roughly categorised into pattern-based, information retrieval-based (IR-based), and classification-based [7,13-15].

Pattern-based systems consist of hand-coded or automatically induced templates derived from sample interaction sentences. The templates, which are sometimes scored for quality, are used to scan text and retrieve any matches. These patterns are usually unable to cover the wide variety of ways with which the interactions can be described in text. For this reason, these methods usually have high precision and lower recall. It is often offered as an argument that experimentally validated relations will be reported several times, thus affording more chance for the interaction to be retrieved [16,17]. Conversely, this approach may only retrieve well known interactions, and as such not be very helpful to a researcher looking for novel interactions in a field that she is familiar with.

On the other side of the spectrum are the methods that consider any co-occurrence of two proteins in a sentence as a possible interaction. This assumption leads to a large number of retrieved interactions, unfortunately with a very low precision rate. A favourite approach of initial systems aiming to construct interaction networks on the fly from user queries, it is an efficient way of allowing the user to browse potential interactions [9,10,18]. More advanced IR-based approaches incorporate interaction detection into the indexing process [11,12]. This allows for fast retrieval of highly detailed information. However, for new types of interactions or entities to be included, the entire collection needs to be re-indexed.

Finally, there are the (mainly supervised) classification-based methods [6,7,13,19-21]. These methods require samples of sentences that are, at the very least, annotated for relevance if not for the full interactions. On the other hand, they are fully automatic, apart from the labelling process. The availability of the standard data, such as AImed [20] and the LLL [22], has allowed for faster development and testing of new algorithms, as well as for comparison across different approaches [6,21,23,24].

What most of these systems have in common is the attempt to fully extract the interaction triples. In this paper, we step back to address a paired down problem: identification of sections of text that describe PPIs (in particular, sentences). In essence, the approach is similar to the latest classification-based methods, in that it employs state-of-the-art kernel classification. On the other hand, it uses bag-of-words [25] representation, which is easier to extract than the parser-based features, that are required for full triple extraction.

Using deep linguistic features increases the complexity of the approach by introducing performance variation with different choice of parser and kernel [26,27]. Although analogous systems have been developed for other domains, such as news, biomedical texts offer particular challenges that need to be addressed with tailored tools [6,28]. Even the detection of the protein names is a difficult problem, because of the high degree of synonymy, polysemy, orthographic variation, and novelty due to protein discovery [29-33]. Protein name recognition is not a necessary step for detection of areas of text that describe interactions [7,34], but for more detailed extraction it is essential [13-15,18,20,35,36].

The approach described in this paper consists of several components that themselves contain parameters that influence the performance of the method. Thus, to study the novelty of this approach we eliminate, as much as possible, reliance on further language processing algorithms. Consequently, by examining a simpler task, we produce results that are not directly comparable with the kernel-based PPI extraction methods described in [26], but are comparable to the baseline results described in [34,37]. However, the approach described here is modular, and can be augmented for use with methods that rely on deep linguistic features.

This paper introduces a method that improves the detection of sentences describing PPIs in biomedical texts. Classification-based methods are usually trained on data labelled by experts. The technique described herein is envisioned as a component of a trainable filtering system, which could be placed on top of a keyword search and could effectively learn from simple annotations provided by a user. For example, a user could indicate whether a sentence describes a PPI or not. Such annotation schemes would be less onerous than ones that require users to label each protein participating in an interaction, and perhaps any other words indicating their relationship. While any pattern recognition or discriminant analysis method could be used for this purpose, the main contribution of this paper is a novel method that enhances the effectiveness of learning from the labelled examples by incorporating semantic information from unlabelled data; and thus reducing the burden on the user.

## Methods

Identification of interactions requires significant biological knowledge. In addition, annotation may also require grammatical expertise, depending on whether entities, interaction identifiers, or even sentence parse trees are considered. While quality labelled data is difficult to obtain in large quantities, unlabelled data is plentiful and freely available in the form of MEDLINE abstracts and full-text open access publications. Semi-supervised learning (SSL) [38,39] is a way to leverage the models trained on labelled data with large amounts of unlabelled data.

A novel approach to semi-supervised learning, where information collected from relevant large datasets, in an unsupervised manner, is incorporated directly into the training kernel was introduced in [34]. The unlabelled corpus is transformed into a matrix of term similarities, which is then projected onto the document vectors causing a rescaling of the labelled training data. In this paper, we extend this method further. Different semantic models can be used to calculate term similarities, each producing slightly different results. In order to take best advantage of the given semantic information, we combine these kernels using probabilistic Multiple Kernel Learning (pMKL).

pMKL is a method that, in single-kernel mode, produces similar results to Gaussian processes (GPs), which are comparable to the popularly used support vector machines (SVMs) [40]. Kernel combinations can be employed using any kernel method, as [14] do with SVMs; however, pMKL is also capable of estimating the best weighted combination of kernels. Whilst in this paper we use combinations of semantic kernels, multiple kernel algorithms can be used to combine various kernels of different feature-types in order to take advantage of several views of a single data set [26].

The rest of this section describes the components that are used in this method: the kernel learning algorithm and the semantic models. Firstly, the general description is given of how the components fit together, then a brief introduction to this particular kernel learning algorithm is provided. This is followed by an introduction to semantic models and then more detailed descriptions of the two models that are used here. This section concludes with a description of the experimental setup and is followed by a section that discusses the results of these experiments.

### Semantic kernel construction and combination

**Figure 1:**
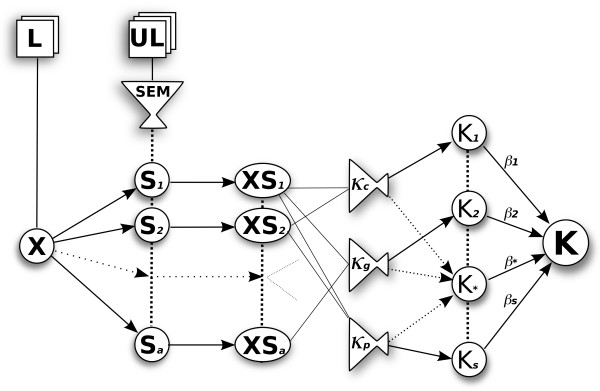
**The overview of the method**. The training data (**X**) comes from the labelled corpus (**L**), while the unlabelled data (**UL**) is transformed using semantic models (**SEM**) to produce smoothing matrices (**S**). The training data is then projected into the semantic subspace (**XS**) and passed into one or more of the available kernel functions. We use cosine (*κ_c_*), Gaussian (*κ_g_*), and polynomial (*κ_p_*) kernels. We combine the resulting kernels with a weighting *β*_*s *_into a single combined kernel (**K**).

The proposed method combines labelled and unlabelled data (semi-supervised learning), by integrating semantic information from unsupervised lexical semantic models trained on a larger corpus, such as the MEDLINE abstracts contained in the GENIA corpus [41]. It is described graphically in Figure 1.

Labelled training data is used for the probabilistic Multiple Kernel Learning (pMKL) classifier training and testing. The training data is represented as a *M *× *N *matrix **X **(where there are M documents and *N *features), and an *M *× 1 vector of training labels.

We then use a semantic model to collect word co-occurrence information. In this paper we compare two such models: Hyperspace Analogue to Language (HAL) [42] and Bound Encoding of the Aggregate Language Environment (BEAGLE) [43]. These give us semantic smoothing matrices **H **and **B**, respectively, to which we interchangeably refer to as **S**. The matrix **H **is a square *N *× *N *matrix, while **B **is *N *× *D*, where *D *is a chosen number of dimensions (defined below in the BEAGLE section).

The semantic information (**S**) is multiplied with the sentence data and thus integrated into the kernel **K **= *κ *((**X **+ *ε*)**S**, (**X **+ *ε*)**S**). A small number *ε *= 0.01 is added to the training data to allow semantic smoothing across the whole feature set. The above approach has the effect of re-introducing the semantic information about the words, that was lost in the bag-of-words representation used to encode the features. Finally, by changing **S **and the kernel function *κ*, we are able to create different kernel matrices, which we then integrate using pMKL. In the following sections we describe pMKL and the semantic word co-occurrence models that comprise this methodology.

### Probabilistic Multiple Kernel Learning

The data integration approach proposed and adopted in the present work belongs to the family of Multiple Kernel Learning (MKL) methods [44-47]. These approaches have recently gained significant attention due to their successful application in bioinformatics and pattern recognition domains [48,49] where multiple information sources are present.

**Figure 2:**
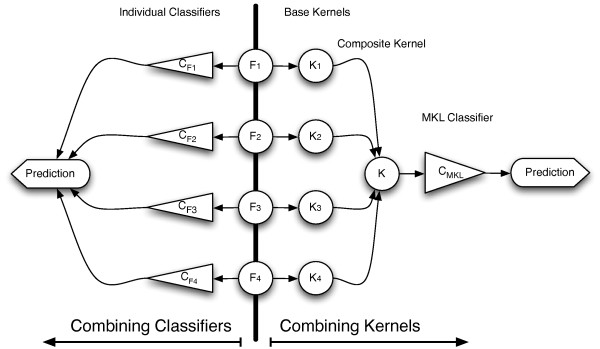
**The intuition behind Multiple Kernel Learning and the differences with Classifier Combination methods**. F*_s _*denote the different feature spaces that are being combined, while the K*_s _*are the different kernels which are contrasted with full classifiers C*_Fs_*, shown on the left-hand side of the figure.

In contrast with past ensemble approaches, such as classifier combination schemes where a separate model was trained on each individual source, MKL is a kernel-based data integration approach that attempts to informatively fuse the information sources *directly *within a single overall model. The intuition behind MKL and the difference from classifier combination methods is graphically depicted in Figure 2.

In this work, we employ the probabilistic MKL approach [50], proposed by [49], which follows a variational Bayesian formalism and results in probabilistic outputs capturing the model's uncertainty in class predictions. Individual semantic kernels, as analytically described in the next sections, are constructed from disparate unlabelled sources and then combined into an overall composite semantic kernel on which a single classifier operates.

The combination follows a parameterised convex linear rule, Equation 1, with kernel combination parameters *β_s _*and individual kernel parameters ***θ***^(***s***) ^for the s semantic kernels (*κ_s_*). Inference of the kernel combination parameters results in identification of an informative fusion of the base semantic kernels and, hence, also acts as a measure of their discriminative power. This will be crucial for selecting appropriate resolution levels for the base semantic kernels later on.(1)

The overall MKL model is a Generalised Linear Model (GLM) [51] employing the multinomial probit likelihood within a variational Bayes approximation as described in [49]. In this work we concentrate on the construction and fusion of the base semantic kernels, which is the focal point of the next sections. The advantage of the adopted methodology is its probabilistic nature which allows a formal way to handle uncertainty.

### Word co-occurrence models

**Figure 3:**
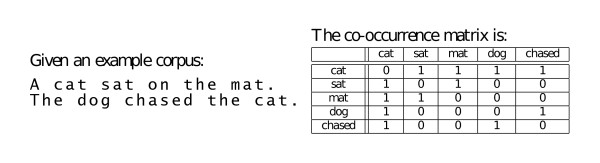
**The vector space representation in a word-based semantic space**. An example of the vector space representation in a word-based semantic space, where the context consists of all the words co-occurring with the target within the sentence. The columns represent the basis words that make up the contexts, while the rows are the target words. In this model the co-occurrence matrix is symmetric. The stop words (*a*, *the*, *on*) are ignored.

Semantic models are representations of word meaning gathered statistically from large amounts of text. In general, they are constructed by considering each individual word in a corpus (referred to as a *target word*, or just *target*) and the text surrounding the word (called the *context*). The set of targets is denoted with *T *, while the set of context words, also referred to as *basis*, is *B*. The basis do not necessarily have to be words. The basis could also be grammatical structures, such as parse or dependency trees [52]. It is important that the basis match the feature type of the kernel. The product is a mapping of words into a multidimensional geometric space, in such a way that distance between the words corresponds to the distance of the word semantics according to their usage in text, Figure 3.

The main purpose of models is to calculate contextual similarity of words, consequently many of the models are constructed on the basis of two principles that reflect this goal. Firstly, in describing semantic models it is often said that "words are known by the company they keep", that is, the target's sense is defined by the surrounding words. Secondly, that this meaning can be learned automatically given enough examples of the usage of a word [53]. These two hypothesis have led to a great number of models, many of which have been validated both in psychological and linguistic experiments [42,43,52,54].

One of the main separating characteristics is the definition of the context used in the generation of the model. For example, Latent Semantic Analysis (LSA) [55] defines documents as contexts. Therefore, the words that are similar are the ones that can be found within same documents. On the other hand, [56] and [52], describe syntax-based models, where the context of the target is a path in the sentence dependency parse tree containing the word. Word co-occurrence models are the ones where the context consists of words immediately surrounding the target, within some specified window. Both HAL [42,57] and BEAGLE [43,58] are word-based models.

Word-based co-occurrence models are generally represented in the vector space. Each word corresponds to a vector whose dimensions are called the *basis*. In general, there exists a mapping between contexts and the basis. If this mapping is 1-to-1, the length of the target vectors is the number of all possible contexts. This can result in high-dimensional space corresponding to the number of unique words in the corpus. To limit the dimensionality and remove some noise, highly frequent function words are usually ignored. There are standard lists of these *stop words *containing most commonly occurring words including pronouns, determiners, and conjunctions. Depending on the final application of the model, the function words usually contain very little information.

#### HAL

**Figure 4:**
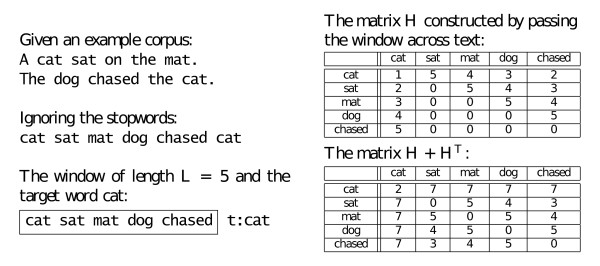
**Construction of a HAL matrix**. Construction of a HAL matrix from a small two-sentence corpus with the window of length L = 5. The stop words (*a*, *the*, *on*) are ignored.

Hyperspace Analogue to Language (HAL) is a semantic model that represents word similarity according to co-occurrence within a window of specific length [42,57,59]. The strength of word co-occurrence is determined by the distance between the two words within the specified window (Figure 4). This has the effect of boosting the similarity between words whose close contexts are the same, while allowing for variation in the phrasing of the context.

The |*T*| × |*T*| HAL matrix, **H***_o_*, is constructed by passing a window of fixed length, *L*, across the corpus. The last word in the window is considered the target and the preceding words are the basis. Because the window slides across the corpus uniformly, the basis words are previous targets, and therefore the set of targets *T *is equivalent to the set of basis *B*, *T *= *B*.

The strength of the co-occurrence between a target and the basis depends on the distance between the two words, *l*, 1 ≤ *l *≤ *L*, within the window. The co-occurrence scoring formula, *L *- *l *+ 1, assigns lower significance to words that are further apart. The overall co-occurrence of a target-basis pair is the sum of the scores assigned every time they coincide within the sliding window, across the whole corpus.

Even though the matrix is square, it is not symmetric. In fact, the transpose of the matrix reflects the co-occurrence scores with the basis that occur within the window of length *L after *the target. Thus **H***_o _*and  together reflect the full context (of length 2*L *- 1) surrounding a target. There are two ways of combining this information so that it would be considered when the distance between targets is calculated. The first way is to concatenate **H**_o _and  to produce a |*T*| × 2|*B*| matrix. The second way is to add the two matrices together . Experimental testing showed that for our kernel combination method that the latter strategy is more effective. This was also the case when HAL was employed for query expansion [60]. Therefore, from now on when we refer to **H **we will assume .

#### BEAGLE

The Bound Encoding of the Aggregate Language Environment (BEAGLE) model [43,58] was proposed as a combined semantic space that incorporates word co-occurrence and word order. It is a word-based method where the context consists of words occurring in the same sentence as the target. Therefore, the set of targets and basis words is the same, and both consist of all unique words in the corpus. The data is stored in a vector space reduced by random mapping. If a context word appears frequently in the same sentence as a target word, its signal will be amplified through addition. Words sharing the same contexts will have strong signals corresponding to the common words.

Random mapping, sometimes also referred to as random projection or random indexing, is a method for reducing the dimensionality of data. For large data matrices, methods based on matrix decomposition such as principle component analysis (PCA) or singular value decomposition (SVD) can lead to heavy computational overheads [61-63]. On the other hand, random mapping provides a computationally efficient method of dimensionality reduction with minimal distortion in the distances between vectors [62]. It has been used for classification and clustering in a variety of applications including image and text [62,64], software quality [65], databases [66], and others [63].

The mapping transforms an *M *× *N *matrix, **X**, into a lower dimensional space by multiplication with a *N *× *D *matrix of random values, **R**. **R **can be constructed by random sampling from any distribution with the mean 0. The normalised rows form a near-orthogonal set of basis. The more dimensions are preserved, the more orthogonal the vectors are. In other words, the matrix **RR**^T ^= **I **+ ε, where *ε *is a small amount of noise that decreases as *D *increases [64].

Random mapping is used in BEAGLE in order to decouple the word vector lengths from the size of the vocabulary, as well as to reduce the vector length in order to allow for more efficient execution of costly matrix operations that are needed to encode word order [43].

The BEAGLE context matrix can be constructed by first building the |*T*| × |*T*| dimensional matrix of co-occurrence frequencies, such as in Figure 3, and subsequently reducing this space by multiplying it by a |*T*| × *D *matrix of random values, **R**. Alternatively, it can be generated sequentially as the corpus is traversed. The latter method is more advantageous in that it allows for an expandable lexicon and it eliminates the need to store and transform the large frequency matrix. Addition of new words through corpus expansion only requires addition of new rows to the matrix.

The number of dimensions *D *is chosen so that it is large enough to ensure that this vector is unique for each target or basis word [58] suggest that multiples of 1024 are an appropriate choice for *D*, and use *D *= 2048 to encode larger corpora. Through empirical testing we found that *D *= 4096 gives us slightly better classification performance.

In this sequential method, each unique word in the corpus is assigned a *D*-dimensional vector of normally distributed random values drawn from the Gaussian distribution . The choice of the standard deviation of  ensures normalised vector lengths. These are referred to as environmental vectors and denoted by **e***_b_*, where *b *is a basis word. The |*T*| × *D *BEAGLE matrix, **B**, where the rows are indexed by target words, is initialised to 0. The text is scanned in order, and for each target word *t_i _*encountered, the context vector  for the current sentence *s_k _*is calculated.  is the sum of the environmental vectors of the basis words, *b_j_*, in the sentence. If we are only considering the contexts, the matrix entry for the target word *t_i _*is the sum of the context vectors gathered form all the sentences *s_k _*such that *t_i _*occurs in *s_k_*, .

### Experimental Setup

In our experiments we want to test the efficiency of combinations of semantic kernels by comparing them to single kernel results. In addition, we want to examine the potential of the weighted combinations of kernels to expand on our knowledge of the semantic methods.

For training data, we use the AImed data set [20], in which the protein entities are annotated and interacting pairs are specified, to judge which sentences contain interactions. The AImed corpus is emerging as standard and is being used in a variety of ways [14,21,23]. It consists of abstracts that contain PPI interactions, and have been annotated for proteins with a scheme that distinguishes the interacting protein pairs. It is, therefore, possible to separate the corpus into a data set that contains positive and negative example sentences. This can be done in two ways. For example, [20,21,23] separate the corpus into pairs of proteins, using the manually annotated protein entities. Interacting pairs are then used as positive training examples, while any two proteins, that occur in the same sentence and do not interact, constitute the negative data.

The other approach, and one which is employed in this paper, is to consider the sentences that contain interactions as positive examples, and the ones that do not, as negative. The reformulation of the problem has several advantages. This task is simpler to annotate than the full PPI, thus allowing for faster production of training data. Feature extraction does not require sentence parsing or preprocessing in a way that may be sensitive to annotation errors. The simpler classification leads to higher precision and recall, but only locates the sentence that describes the PPI and not the exact interacting pair. Thus while it is not fully automated, it might be more useful in a curation pipeline where the results need to be checked by humans [28]. Using the provided sentence segmentation, the data set contains 614 positive and 1355 negative sentences. All sentences are included regardless of the number of annotated proteins contained within.

**Figure 5:**
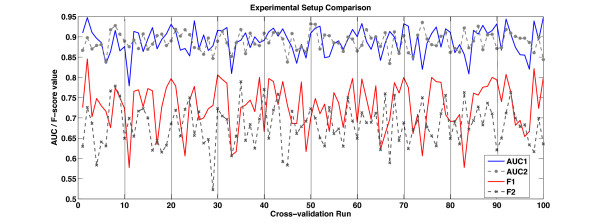
**Comparison of experimental settings**. This graph represents the AUC (higher pair of lines) and the F-score (lower pair of lines) results for two different experimental setups. In the first setup (AUC1, F1), all of the sentences from an abstract are either in training or test data within a fold of the cross-validation experiment. In the second setup, the sentence vectors are randomised first and then cut into cross-validation folds. The experiment shows 10 runs of randomised 10-fold cross-validation experiments. The vertical bars demonstrate the separations between the runs. For the first experiment *F*_1 _= 0.7300 ± 0.0058 and *AUC *= 0.8902 ± 0.0033, while for the second experiment *F*_1 _= 0.6808 ± 0.0051 and *AUC *= 0.8886 ± 0.0023. The t-test shows there is no significant difference between these experimental setups (*p *= 0.9640 for the F-scores and *p *= 0.5467 for the AUC).

When AImed data is used with the syntactic features, for example in [23,67], it is usually applied with the original 10-fold cross-validation (10 × 10 cv) data split provided by the dataset authors [20]. We can see from Figure 5, which demonstrates 10 different runs of a 10 cv experiment, that there can be great variations between the performance of an algorithm on different randomisations of data.

Therefore, it is more rigorous to run 10 different randomisations and all experiments are performed using ten times ten cross-validation (10 × 10 cv). In this way the training data is randomised, separated (as closely as possible) into ten equal parts. Nine of these parts are used for training and one for testing. This procedure is repeated with 10 different randomisations of the original data, providing 100 values for significance testing. In Figure 5, we show an illustration of two methods of data segmentation. In one method, the data is segmented so that no sentences in the test data come from the same abstract as a sentence in the training data. The abstract order is first randomised, the data is split into training and test portions as described above. The sentences are then further randomised within their set. In the other method, the sentences are randomised without observing the abstract of origin. In this particular experimental setup, the choice of randomisation technique provides no statistical difference across all of the experiments. The former method of sentence randomisation is also coupled with per-fold parameter tuning, while in the latter a single parameter is chosen for all of the folds. This may account for slightly better performance and the higher variance of the more stringent training-test data split. Significance testing is performed using a version of Student's t-test designed for 10 × 10 cross-validation [68].

We extract our training data from the AImed corpus in a manner similar to the vector space example in Figure 3. Each sentence is scanned and all the stop words [69] are removed. Each word is reduced to lowercase, any symbols or numbers are discarded, and the words are truncated to 10 letters [7]. Protein names are substituted by placeholder strings *PTNGNE *concatenated to the number of the protein within the sentence. This leaves 3,084 unique features. A sentence is then represented as a vector indexed by the unique features in the corpus. The number of times each feature occurs in the sentence is recorded in the vector. The anonymisation of proteins is likely to be one of the factors that minimises the effect of data randomisation methods (see Figure 5). The other factor is that sentence structure, and thus to some degree the authorship style, is disregarded.

The words in the corpora that were used as unlabelled data, GENIA and the subset of the Biomed Central open access articles [70] (OAA), are processed in the same way. GENIA is annotated for protein names, the OAA is not. So, for compatibility reasons, we have processed OAA with the Lingpipe sentence segmentation and named entity recognition software trained on GENIA. We used the *protein molecule *annotation as the indicator of protein presence. The OAA dataset also differs from AImed and GENIA in that it consists of full text articles, thus the results consist of a smaller portion of text and are described in a different, more detailed way. Similarities are created only for the words that occur in the training data. The HAL matrix is created as described in the previous section and in Figure 4, except that only the unique features from AImed are considered as targets and basis. This leads to a sparse *N *× *N *matrix **H**, which is then multiplied with **X**. On the other hand, for the BEAGLE matrix we still only consider AImed training features as targets; however, the bases consist of all words that co-occur in the sentences with these targets. For GENIA there are around 12,000 basis, and for OAA only the first 30,000 basis are considered, but the random projection technique keeps the BEAGLE matrix size consistent at *N *× *D *dimensions, where *D *= 4096.

We measure the efficiency of classification using the AUC and F-score measures. The AUC is the area under the receiver operating characteristic (ROC), which depicts the true positive rate vs. the false positive rate of a classifier's testing output. The closer the AUC is to 1, the better the classification results. The F-score is often used in evaluating natural language processing tasks. It is a balanced measure of precision (*P*) and recall (*R*). Here we use . The error is defined as the percentage of testing points that were wrongly classified.

**Figure 6:**
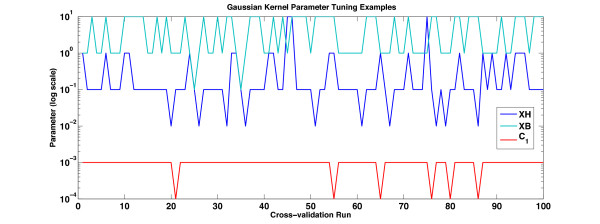
**Kernel Parameter Estimation**. This graph shows the parameters that were chosen for some of the experiments in this paper. The top two lines describe experiments with the OAA dataset whose results are shown in Table 1. While the third line describes the parameters chosen for the combined kernel **C**_1 _from Table 2.

Finally, the pMKL algorithm has no parameters akin to the SVM regularisation parameter. The only parameters that required tuning were the Gaussian kernel parameter and the HAL window size parameter. The Gaussian kernel parameter that produces the highest AUC with both HAL and BEAGLE kernels is determined using 1 × 3 cross-validation at each fold of the 10 × 10 cv experiment (Figure 6). The range of values examined are the powers of 10 between 10^-5 ^and 10. For the combinations of composite HAL kernels the preferred values tended towards the smaller parameters, for plain data the parameter chosen was 0.1 for 99 of the 100 folds, while for data transformed by a single HAL or BEAGLE kernel, the values ranged in the set (0.1, 1, 10). The approach of tuning parameters at each fold is time consuming, but 1 × 3 cv performs just as well as 2 × 5 cv, in this experiment. There is also no statistical difference between choosing the best result out of several parallel 10 × 10 cv experiments, each run with a particular assigned kernel parameter, and the above per-fold tuning method. The polynomial kernel parameter is 2.

## Results and Discussion

The purpose of the experiments in this paper is to verify that using combinations of multiple semantic kernels can improve classification performance. We do this with the simplest possible features, in order to avoid introducing further complexity. As a result, it is difficult to compare the results to full PPI extraction tasks, so we provide single kernel baseline results. In the general, the pMKL algorithm produces results comparable to the SVM, but without the need to tune the extra margin parameter. Depending on the task, features, kernel, kernel parameter, and margin parameter choice the pMKL, GPs, and the SVM might slightly, but significantly outperform each other, but in general will provide similar results [40]. This section is divided in three parts. In the first part, we provide the baseline results using plain and semantic kernels. Secondly, we examine many fixed combinations of the basic kernels, and report the best combinations. In the final part, we examine the effectiveness of the pMKL's ability to estimate the best weighted sum of the kernels, by observing the changes in the predictive likelihood. This estimation is done without observation of the true labels of the test data, and therefore might not lead to the optimal F-score or AUC.

### Single kernel results

The baseline for the evaluation of pMKL is provided through single kernel experiments, the results of which are provided in Table 1. These results are consistent with the semantic kernel experiments performed with the GP classifiers in [34]. The ultimate baseline is provided by using pMKL with a plain Gaussian kernel, which produces higher F-score and AUC than the cosine and polynomial kernels.

**Table 1:** Results of the pMKL single kernel experiments

Kernel	F-score	Error	Precision	Recall	AUC
**C**_0_: **X**^a^	0.7300 ± 0.0058	17.6878 ± 0.3158	0.6893 ± 0.0072	0.7828 ± 0.0079	0.8902 ± 0.0033
**XH**^b^	0.7060 ± 0.0060	18.3453 ± 0.3203	0.6989 ± 0.0074	0.7210 ± 0.0084	0.8899 ± 0.0031
**XB**^b^	0.6567 ± 0.0080	20.6249 ± 0.4113	0.6759 ± 0.0086	0.6501 ± 0.0113	0.8776 ± 0.0035
**XH**^c^	0.6921 ± 0.0056	18.5716 ± 0.3200	0.7113 ± 0.0072	0.6808 ± 0.0077	0.8888 ± 0.0029
**XB**^c^	0.7267 ± 0.0052	17.2958 ± 0.3087	0.7117 ± 0.0070	0.7490 ± 0.0071	0.9000* ± **0.0028**

^a ^The original data with the Gaussian kernel.

^b ^The data smoothed with the HAL and BEAGLE (Gaussian) matrices created from GENIA dataset.

^c ^The data smoothed with the HAL and BEAGLE (Gaussian) matrices created from the OAA dataset.

* Statistically significant (*p *= 0.0038) compared to kernel **C**_0_.

We then add the Gaussian semantic kernels created using HAL and BEAGLE. We find that with pMKL, unlike with GPs, the smoothing using the HAL matrix produces a slight reduction in AUC over the plain Gaussian kernel, although this is not statistically significant. While there is little difference between the results produced with the HAL kernel created from Genia or OAA, the larger data set produced significantly better results when applied with the BEAGLE method.

In these experiments **H **was constructed from *l *= 1 which was shown to lead to the best results with this data and GPs [34]. Under the speculation that more data is better than hand annotated data, we proceed with multiple kernel experiments using the OAA dataset.

### Multiple kernel results

We perform two types of multiple kernel experiments. In the first kind we evaluate uniform compositions of multiple kernels, we then estimate combinations of different kernels in order to gain insight into their predictive properties. Many different combinations of semantic kernels could be formed, so the following experiments are illustration of possible uses.

Table 2 shows that combinations of kernels can lead to a statistically significant increase in the AUC. As the parameter tuning was performed with the observation of the maximum AUC, the results reflect that. For experiments where the F-score is the primary concern, the tuning should be performed by observing the highest F-score. While Figure 5 demonstrates the general trend of the two measures is similar, the tuning strategy can make a difference in the outcome. The kernel combination **C**_1 _is the uniform weighting of the HAL matrices at different window lengths together this weighting is equivalent to . We find that there is no difference in performance over smoothing with the single kernel **H**_*l *= 1 _from Table 1. The kernel combination **C**_2 _is the uniform sum of combined HAL matrices , where . This combination contains redundant information over **C**_1_, but this strategy provides only a decrease in performance. However, **C**_3_, which is a combination of different views of the data using different kernel types and combinations of both GENIA and OAA **H**_*l *= 1 _smoothing provides an increase in performance. This indicates that the performance gain is best achieved when combining kernels that contain diverse information or at least diverse views of that information.

**Table 2:** Results of the pMKL multiple kernel experiments with fixed weights

Kernel	F-score	Error	Precision	Recall	AUC
**C**_1_	0.7039 ± 0.0054	17.5614 ± 0.3378	0.7381 ± 0.0059	0.6790 ± 0.0080	0.8881 ± 0.0029
**C**_2_	0.7052 ± 0.0054	18.3698 ± 0.3337	0.7012 ± 0.0063	0.7155 ± 0.0076	0.8838 ± 0.0031
**C**_3_	0.7359 ± 0.0045	16.8581 ± 0.2934	0.7156 ± 0.0063	0.7631 ± 0.0063	0.9092* ± **0.0023**

**C**_4_	0.6633 ± 0.0080	19.0861 ± 0.3507	0.7301 ± 0.0078	0.6242 ± 0.0119	0.8883 ± 0.0029

**C**_1_: Uniform sum of Gaussian kernels from HAL matrices with window lengths *l *1 through 10 (OAA).

**C**_2_: Uniform sum of Gaussian kernels from HAL matrices with window lengths *L *1 through 10 (OAA).

**C**_3_: Uniform sum of 6 Gaussian, cosine, and polynomial kernels using HAL **H**_*l *= 1 _from both OAA and GENIA.

**C**_4_: Estimated sum of Gaussian kernels from HAL matrices with window lengths *l *1 through 10 (OAA).

*Statistically significant (*p *= 0.0016) compared to kernel **C**_0_.

### Estimating the kernel weights

Although the best results come from fixed kernel weights, we can gain significant insight into the predictive quality of the data by exploiting the pMKL kernel weight estimation property.

In particular, we are interested in examining the properties of HAL matrices. These matrices are composites and each matrix **H **created with context length *L *can be considered a combination of *L *matrices, such that . Therefore, in addition to the right choice of kernels and kernel settings we need to make the right choice of *L*. There is also a dispute over the weighting function (*L *- *l *+ 1); for example, [71] found that using a uniform weighting as opposed to a decaying one produces better search query expansion results.

**Figure 7:**
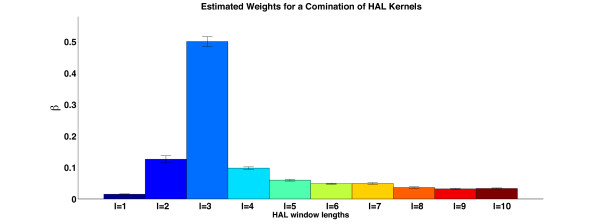
**The weights assigned by pMKL to words at different distances from the target word**. The bars represent the kernels constructed from HAL matrices with, from left to right, *l *= 1 to *l *= 10.

Figure 7 shows the estimated weights for kernels constructed with **XH***_l _*for *l *= 1 ... 10 (**C**_4_, in Table 2). The assigned weightings closely mirror the sparsity of the HAL matrices. Matrices 2 and 3 have the lowest sparsity, and while the contribution of *l *= 1 seems to be underestimated, *l *= 3 seems to be overestimated. This would indicate that, perhaps, a scheme weighted by the information stored in matrices representing various window lengths would lead to best performance when applying the HAL algorithm to various tasks. The AUC (0.8883 ± 0.0029) is slightly higher than the uniform combination of these kernels while the F-score (0.6633 ± 0.0080) is significantly lower than in uniform combination of these kernels (**C**_1_, in Table 2). Due to the computationally intensive nature of this experiment the parameters for each of the kernels was set at 1, which is one of the parameters the cross-validated tuning approach favoured for **XH**_*l *= 1 _(Figure 6).

## Conclusions

This paper describes a smoothing approach, which is similar to the methods using semantic kernels created from WordNet [72] or Wikipedia information [73,74]. However, this method provides a domain-independent alternative, by using automatically derived semantic information for classification. It also gives an application-based way of evaluating the quality of word co-occurrence matrices, which is a difficult task usually requiring specialised human judgements.

The results presented in this paper show that using combinations of kernels can lead to significant improvement in both F-score and AUC. In addition, we are able to use pMKL kernel weight estimation for kernel selection as well as for gaining important insights into the quality and linguistic properties of the data. This is an introduction to this approach, which uses simple features that do not require dependency parsing, and thus is not directly comparable to the full extraction methods that are popularly used with PPI data sets. In the future we will investigate this method with semantic models that are compatible with dependency-based features, and kernels.

## Competing interests

The authors declare that they have no competing interests.

## Authors' contributions

TP and MG designed the semantic kernel method. TD and MG designed the pMKL approach, which was implemented by TD. TP implemented the semantic kernel method and ran the experiments in this paper. All authors read and approved the final manuscript.
